# Predict drug sensitivity of cancer cells with pathway activity inference

**DOI:** 10.1186/s12920-018-0449-4

**Published:** 2019-01-31

**Authors:** Xuewei Wang, Zhifu Sun, Michael T. Zimmermann, Andrej Bugrim, Jean-Pierre Kocher

**Affiliations:** 1 Department of Health Sciences Research, Mayo Clinic, Rochester, MN USA; 2 Silver Beach Analytics Inc, St Joseph, MI USA; 3 Present address: Bioinformatics Research and Development Laboratory, Genomic Sciences and Precision Medicine Center, Medical College of Wisconsin, Milwaukee, WI USA

**Keywords:** Pathway activity, Drug sensitivity, Precision therapy, Machine learning, Cancer, Pharmacogenomics

## Abstract

**Background:**

Predicting cellular responses to drugs has been a major challenge for personalized drug therapy regimen. Recent pharmacogenomic studies measured the sensitivities of heterogeneous cell lines to numerous drugs, and provided valuable data resources to develop and validate computational approaches for the prediction of drug responses. Most of current approaches predict drug sensitivity by building prediction models with individual genes, which suffer from low reproducibility due to biologic variability and difficulty to interpret biological relevance of novel gene-drug associations. As an alternative, pathway activity scores derived from gene expression could predict drug response of cancer cells.

**Method:**

In this study, pathway-based prediction models were built with four approaches inferring pathway activity in unsupervised manner, including competitive scoring approaches (*DiffRank* and *GSVA)* and self-contained scoring approaches (*PLAGE* and *Z-score)*. These unsupervised pathway activity inference approaches were applied to predict drug responses of cancer cells using data from Cancer Cell Line Encyclopedia (CCLE).

**Results:**

Our analysis on all the 24 drugs from CCLE demonstrated that pathway-based models achieved better predictions for 14 out of the 24 drugs, while taking fewer features as inputs. Further investigation on indicated that pathway-based models indeed captured pathways involving drug-related genes (targets, transporters and metabolic enzymes) for majority of drugs, whereas gene-models failed to identify these drug-related genes, in most cases. Among the four approaches, competitive scoring (*DiffRank* and *GSVA)* provided more accurate predictions and captured more pathways involving drug-related genes than self-contained scoring (*PLAGE* and *Z-Score*). Detailed interpretation of top pathways from the top method (*DiffRank*) highlights the merit of pathway-based approaches to predict drug response by identifying pathways relevant to drug mechanisms.

**Conclusion:**

Taken together, pathway-based modeling with inferred pathway activity is a promising alternative to predict drug response, with the ability to easily interpret results and provide biological insights into the mechanisms of drug actions.

**Electronic supplementary material:**

The online version of this article (10.1186/s12920-018-0449-4) contains supplementary material, which is available to authorized users.

## Background

Determining the responses of individual patients to drugs has become a critical task in the practice of personalized medicine. Experimental efforts have been undertaken to directly measure drug response of the cells extracted from patients’ cancerous tissues, including in-vitro and in-vivo models [1]. While such experimental approaches capture biological characteristics of patients’ tumor, the high-cost and time-consuming operations render them hardly scalable in practice.

With the advance of high-throughput genomic technologies, pharmacogenomics is becoming a powerful approach to determine individuals’ responses to drug therapies [2]. Typically, studies generate molecular profiles (i.e. SNPs, gene or protein expressions, etc) from cell lines, measure cellular responses to drugs, and then develop computational models to predict drug responses [3]. These computational models could be applied to identify molecular determinants of drug response and further stratify patient population for given drug therapies, with the assumption that cell line models yield clinical relevance [4]. For example, earlier efforts on NCI-60 panels [5] have highlighted specific genetic aberrations as drug targets or biomarkers informative of drug response. For instance, BRAF and EGFR mutations are currently used to predict response to specific kinase inhibitors [6]. Later, studies like Cancer Cell Line Encyclopedia (CCLE) [7], Genomic Drug Sensitivity of Cancer (GDSC) [8] and GSK panel [9] have extended to large-scale collection of cell lines with drug responses and more molecular data types. These large cell line datasets provide a more comprehensive representation of the genomics variability observed in tumors providing new means to identify novel drug targets or drug response biomarkers. These large datasets can also be used to develop computational models to predict drug responses. For instance, CCLE and GDSC have been used to evaluate the robustness of linear prediction models [10], develop novel computational approaches identifying combinatorial biomarkers of drug response [11] and validate prediction models with genomic and chemical features [12].

Exploring these data-resources can help uncover new drug mechanisms and further personalize drug therapies. Currently, most of the computational models to predict drug sensitivity of cancer cell lines involve gene-level features like gene expression [3]. However, gene level features have been reported as having limited reproducibility across independent studies and challenges to biological interpretation [13]. There is growing evidence that drug responses could be modulated by the concerted behavior of multiple genes, instead of individual genes [14]. Pathway (or gene-set) based approaches can help to take into account such coordination of genes, reduce model complexity and increase explanatory power of prediction models [15]. In fact, pathway approaches have been successfully applied in disease classifications [16, 17] by aggregating gene expressions into pathway-level activities used for prediction. In the context of drug sensitivity, such pathway-based approach may also help improving predictions. While gene-level models have been validated and compared [10, 18], the performance of pathway-based approach in this context is yet to be investigated and validated.

In this study, we investigate four representative approaches to score pathway activities solely based on gene expression data alone. Specifically, these four approaches were compared based on 24 drugs from CCLE dataset [4], in term of their performance to predict drug response and the ability to recapitulate target-related pathways. For each approach, sample-wise pathway activity scores were first calculated for cell lines, and then were used as inputs in Elastic net [19] models to predict drug responses.

## Methods

### Data sets

Raw gene expression and drug response data (IC_50_) were collected from the CCLE for 24 drugs. Specifically, raw gene expression data (Affymetrix cel files) was first extracted and normalized with Bioconductor *Affy* package (MAS5 algorithm) and then log-transformed. For genes with multiple probesets, the optimal probeset was then determined using R package *jetset* [20]. For each drug, IC_50_ values are log-transformed for downstream analysis. Only the cell lines with both gene expression and response data are used to build prediction for each drug. Note that, the number of cell lines varies with drugs, because some cell lines may not have response data for all drugs.

Canonical pathways are collected from MetaCore pathway knowledge database, including pathways defined for specific diseases, biological process or certain stimulus. Our analysis is restricted to the 1410 pathways consisting of [5, 200] member genes.

### Modeling workflow

**Fig. 1:**
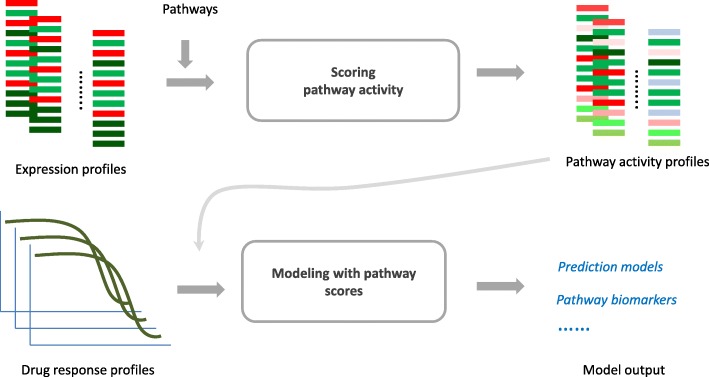
Pathway-based modeling workflow with two major steps (inferring pathway activity and building models with pathway activity in samples)

Pathway-based models integrate gene expression with pre-defined pathways to predict drug response and identify associated mechanistic biomarkers. The modelling process consists of two major steps (Fig. 1): (1) scoring pathway activities based on gene expression profiles from individual cell lines; (2) building prediction models of drug response with pathway activity scores as input features.

### Pathway activity scoring approaches

First step in our model workflow is to score pathway activities for cell lines based on their gene expression profiles. Four unsupervised pathway scoring approaches were looked at in our study. For a given pathway, *PLAGE* method [21] decomposes expression data of member genes and extracts meta-feature by singular vector decomposition (SVD). *Z-Score* approach [17] first standardizes gene expression data and aggregates z-scores of member genes into a combined Z-score as pathway activity*. GSVA* [22] first uses non-parameter kernel estimation to calculate gene-level statistics (evaluating whether a gene is lowly or highly expressed in individual samples) and then aggregates gene statistics into pathway activity in a similar manner with GSEA. Here we introduce a new ranking-based approach (called *DiffRank*) to score pathway activities in individual samples. For a given sample, genes are first ranked in the descent order of their expression levels, and then the rankings of member genes are aggregated into a single score for each pathway. *DiffRank* is straightforward to be calculated on one single sample and do not require multiple samples or phenotype information. For one given pathway, *DiffRank* looks at the difference of average ranking between member and non-member genes in a pathway, and is defined as below:

$$ Diff\ Rank=\frac{1}{n_1}\sum \limits_{i=1}^{n_1}{r}^1-\frac{1}{n_2}\sum \limits_{j=1}^{n_2}{r}^j $$

Where *n*_*1*_ and *n2* are the numbers of member and non-member genes of a given pathway, respectively. Likewise, *r*^*i*^ and *r*^*j*^ represent the rankings of individual member and non-member genes based on their expression levels in samples.

Note that these four pathway scoring approaches could be grouped into two categories. Specifically, both *DiffRank* and *GSVA* score the pathway activity as a function of genes inside and outside pathways, analogue to the competitive gene-set analysis. In contrast, *PLAGE* and *Z-Score* consider only the genes inside pathways, analogue to the self-contained gene-set analysis. *DiffRank* is implemented from scratch and all the other three approaches are adopted from the *gsva* package in Bioconductor.

### Building prediction model of drug response

Once pathway activity scores are generated for cell lines, various machine learning models could be applied to predict drug response. We noticed that most individual pathway-level or gene-level features were modestly correlated to drug response for most drugs (data not shown). For such datasets, machine learning models with regularization (i.e. Elastic net) have proven promising to achieve better predictions, as demonstrated by model choices in previous studies [7, 8] and the recommendations from a recent effort assessing models for drug sensitivity prediction [18]. As such, Elastic net algorithm (from R package “glmnet”) is used to build the prediction models, and other machine learning algorithms are not considered in this study. The optimal parameters of predictive model are determined through 10-fold cross validations. In particular, a grid of 2500 settings of elastic net parameters (α: 10 settings in [0.2, 1]; λ: 250 settings in [*exp*^*− 6*^, *exp*^*5*^] was searched in cross validations.

## Results

### Overview of overlaps and correlations among pathway member genes

**Fig. 2:**
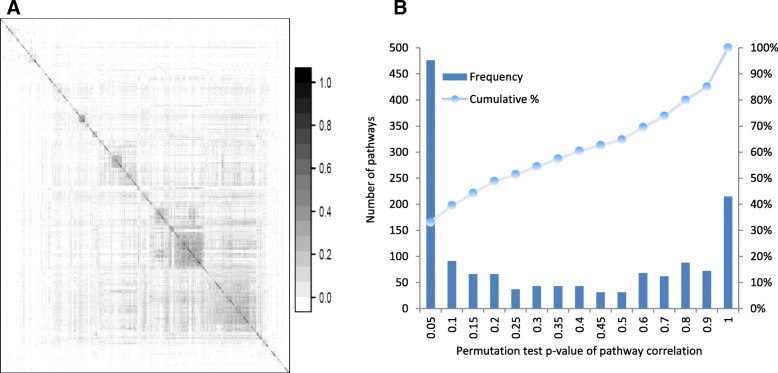
Pathway overview: (**a**) heat map of Jaccard Index indicating the overlaps of pathway member genes; (**b**) histogram regarding the *p*-values of pathway correlations (MACC) from permutation test

The overlaps of member genes were first explored for all 1410 pathways. Specifically, Jaccard Index was calculated to measure the overlap between two pathways. The value of Jaccard index ranges from zero (for pathways without overlapping genes) to one (for identical pathways). Figure 2 (panel A) shows the heatmap of Jaccard index values for all pathway pairs. As shown, most pathway pairs have small Jaccard index, indicating the slight overlaps among these pathways. There are handful blocks of pathways with relatively bigger overlaps, though their Jaccard index values are fairly modest (less than 0.4). This suggests that the Metacore pathways are generally specific and do not have much redundancy to other pathways, which would help ease the concerns caused by overlapping genes in pathway analysis.

We further explored the correlations of member genes within individual pathways. For each pathway, Pearson correlation coefficients were calculated for all pairs of member genes. The median of absolute correlation coefficients (MACC) was taken as an overall measure of pathway member gene correlations. Then permutation test was performed to determine if member genes within one pathway have higher correlations than by chance. Specifically, gene expressions of cell lines were randomly shuffled for 1000 times to generate a vector of random median correlations (MACC). For each pathway, the statistical significance of real MACC is then determined by comparing to random MACCs. For example, the *p*-value would be zero if all random MACCs are smaller than real MACC. The results (Additional file 1) shows that ~ 40% pathways (565 out of 1410) have p-value less than 0.1 as shown in Fig. 2 (panel B). Interestingly, many of the most significant pathways are indeed relevant to cancer mechanisms, such as cell cycle, DNA damage, apoptosis, P53 activation, and translational process with CFTR etc. In contrast, many least significant pathways tend to be defined for other conditions (i.e. asthma, diabetes, cardiovascular) or biological processes (i.e. nicotine regulation, neurophysiological process). This observation is concordant to the notation that pathways are generally condition-specific, since only cancer cell lines are used to generate the CCLE dataset.

### Prediction performance of pathway-based models

**Fig. 3:**
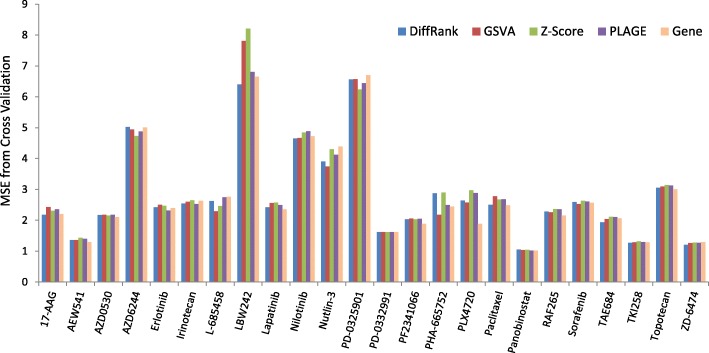
Prediction performance of pathway-based models for 24 CCLE drugs

Pathway approaches have been applied to identify disease biomarkers and patient stratification. Critical to these approaches is to characterize pathway activity with a quantitative score aggregated from gene expression data. Four unsupervised pathway scoring approaches are compared in our study, including *PLAGE*, *Z-Score*, *GSVA* and *DiffRank*. All four pathway score metrics were first calculated for all cell lines with gene expression data in CCLE. For each drug, cell lines with both response data (IC50) and pathway activity scores were used to build the prediction models. In particular, elastic net model was trained for pathway activity scores with 10-fold cross validation to determine optimal parameters yielding minimal mean square error (MSE). As a comparison, elastic net models were also trained with gene level expression data for all 24 drugs. Figure 3 provides the prediction performance (MSE) from cross validation for pathway-based models and gene-level models on all the 24 drugs in CCLE.

As shown in Fig. 3, *DiffRank* performs the best for 9 drugs and the second best for 8 drugs, whereas *GSVA* has best prediction for 7 drugs and the second best prediction for 5 drugs. *Z-Score* and *PLAGE* have best prediction performance for the rest 8 drugs, but poorest performance for 16 drugs. The superior performance of competitive scoring over self-contained score suggested that incorporating both member and non-member genes may better capture the variations of pathway activities among individual samples. Comparing to gene-level models, at least one pathway-based model perform better for 14 of the 24 drugs. Take *DiffRank* as an example, it outperforms gene-level models for 11 drugs. Meanwhile, gene-level models perform the poorest for three drugs (Nutlin-3, PD-032991 and ZD-6474). For these drugs, pathway-based models could be promising alternatives for predicting their sensitivity on cancer cells.

### Identification of pathways involving drug-related genes

Elastic net identifies the features with non-zero weights as important features predictive of cellular response to drugs. In order to evaluate the biological relevance of important features identified from elastic net models, we have collected the drug-related genes (targets, transporters and metabolic enzymes) from commercial and public resources (i.e. MetaCore, DrugBank and original CCLE publication) for all drugs (Additional file 2). We further investigated whether pathways involving these drug-related genes could be captured by pathway-based models.

**Fig. 4:**
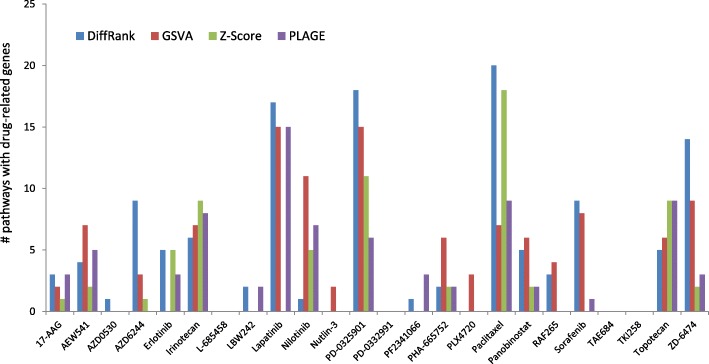
Number of pathways involving drug-related genes for 24 drugs

Figure 4 provides the number of pathways (involving at least one drug-related gene) from each approach for these drugs. We can see that all pathway approaches identify relevant pathways for many drugs. In particular, *DiffRank*, *GSVA*, *Z-Score* and *PLAGE* identified pathways involving drug-related genes for 18, 16, 12 and 15 drugs, respectively. Consistent with the observations of model prediction performances, competitive scoring approaches (*DiffRank* and *GSVA*) tend to identify drug-related pathways for more drugs than self-contained approaches (*Z-score* and *PLAGE*). However, none of these approaches identify drug-related pathways for three drugs, including PD-0332991,TAE684 and TKI258.

We also looked into the genes identified by the gene-level models described earlier, against the drug-related genes. It turns out that these gene-level models identified only one target gene for three drugs (Lapatinib, RAF265 and TAE684), one enzyme gene for Sorafenib, but could not capture any drug-related genes for all the other 15 drugs. This indicates gene expression alone can barely identify drug-related genes in majority of cases, which corroborates the notion that the activities of many targeted proteins are not necessarily reflected by their gene expressions.

### Pathways recapitulating known drug mechanisms

**Fig. 5:**
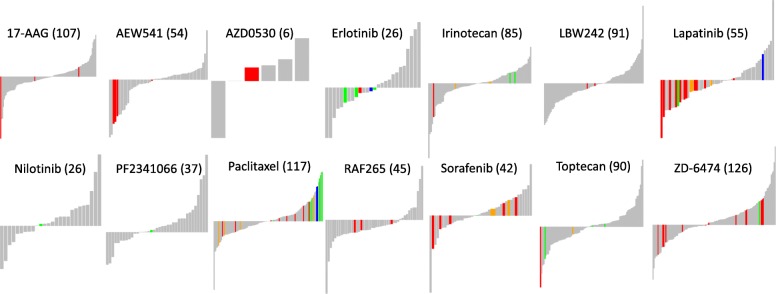
Waterfall plots for the important pathways identified by *DiffRank* models. Pathways are ranked based on the coefficients in models and the numbers of identified pathways are given after drug names

Among the four pathway scoring approaches, *DiffRank* tends to achieve better predictions for more drugs and is more capable of capturing pathways involving drug-related genes. For the 24 CCLE drugs, *DiffRank* performed either best or second best for 17 drugs, and identified pathways with drug-related genes for 18 drugs, with 14 drugs in common. We then investigated biological relevance of important pathways (with non-zero coefficients in models) identified by *DiffRank* for these 14 drugs. In Fig. 5, all identified pathways are ranked based on their coefficients and the ones involving drug-related genes are highlighted with colors as following: (1) *Blue* for pathways with target genes and at least one transporter or metabolic enzyme; (2) *Red* for pathways involving target genes only; (3) *Orange* for pathways involving metabolic enzymes only; (4) *Green* for pathways involving transporter genes only. Please see Additional file 3 for the data used to generate Fig. 5.

As demonstrated, *DiffRank* identified top pathways involving drug-related genes (particularly drug targets) for several drugs, including 17-AAG, AEW541, Irinotecan, Topotecan, Lapatinib, Sorafenib, Paclitaxel and ZD6474. Because of space limitation, we would not discuss each pathway, but rather summarize and highlight a few advantages of pathway models with concrete examples. First, pathway models could identify pathways involving multiple targets. Taking Lapatinib as an example, this drug is a dual inhibitor of EGFR and ERBB2 (or HER2) [23], and was initially approved for treating breast cancer with over-expression of HER2. Gene-level model only identified ERBB2 but not EGFR (see Lapatinib in Additional file 4). In contrast, pathway models trained with CCLE data successfully identified a few top pathways involving both ERBB2 and EGFR, including “anti-apoptotic action of ErbB2 in breast cancer” (see Additional file 5), “ERBB family signaling”, “mitogenic action of ErbB2 in breast cancer” and “EGFR signalling via small GTPase”.

**Fig. 6:**
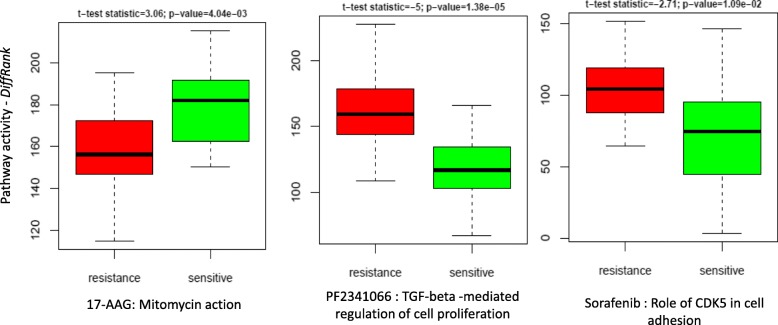
The pathway activity (*DiffRank*) of example pathways between sensitive and resistance cell lines. For each drug, 20 cell lines with lowest and highest IC50 are chosen for sensitive and resistant group, respectively. T-test statistics and p-values are also provided for the pathway activity difference

We also found that pathway models could capture simultaneously both targets and biomarkers. For instance, the pathway “Mitomycin action” is ranked at top one for 17-AAG (see Additional file 3). As shown in pathway map (see Additional file 6), this pathway involves not only one target (CHEK1) of 17-AAG, but also one more gene (NQO1) recently identified as a biomarker for this drug in other studies [7, 10]. Indeed, NQO1 was also ranked at 1st by gene-level models (see 17-AAG in Additional file 4). Studies showed that NQO1 activates 17-AAG [24] and also sensitizes the response of malignant melanoma cells to 17-AAG [25]. This is consistent with the pathway activity of “Mitomycin action” observed on CCLE cell lines, namely this pathway has higher activity scores in cell lines sensitive to 17-AAG (left panel in Fig. 6).

In the meanwhile, pathway models also captured relevant mechanisms for drugs with similar mechanisms. For example, both Irinotecan and Topotecan are toxic chemotherapies and share same mechanism through inhibiting topoisomerase I (TOP1). Pathway models identified one common pathway “Cell Cycle- Chromosome Condensation” involving TOP1 for both drugs. Specifically, this pathway ranked 2nd for Topotecan and 6th for Irinotecan (see these two drugs in Additional file 3). siRNA knockdown of one chromosome condensation regulator reduced cell proliferation, caused cell-cycle arrest, and increased apoptosis [26]. Other studies also showed that drugs targeting topoisomerases inhibit chromosome condensations [27], suggesting the inhibition of chromosome condensation is potentially part of underlying mechanisms of Irinotecan and Topotecan.

Pathway models also identified some pathways without drug targets, but known to be relevant to drug responses. For example, the pathway “Normal and pathological TGF-beta-mediated regulation of cell proliferation” ranked at 2nd for PF2341066 (Crizotinib). Researcher has found that activation of TGF-beta receptor signaling confers to the resistance to PF2341066 [28]. Interestingly, this was confirmed with the elevated activity in CCLE cell lines resistant to this drug (middle panel of Fig. 6). Another example came from the pathway “Role of CDK5 in cell adhension” ranking at 7th for Sorafenib. The activity of this pathway is significantly lower in sensitive CCLE cell lines as shown in the right panel of Fig. 6. A recent study discovered that knockdown of CDK5 can inhibit tumor growth in mouse model [29]. Indeed, a more recent study showed that inhibiting CDK5 improved the sensitivity to Sorafenib-induced tumor suppression in xenografts of hepatocellular carcinoma cells [30].

## Discussion

In this study, we evaluated different unsupervised pathway activity inference approaches for predicting drug sensitivity of cancer cell lines. Our study highlighted the ability of pathway-based models to reveal drug mechanisms, along with prediction performance comparable to gene-based models. Also, pathway-based approach could help generate testable hypotheses by looking at the difference of pathway activity scores between sensitive and resistance cell lines, as demonstrated by the cases in Fig. 6.

A crucial step in pathway-based modelling is to convert gene expression profile to pathway activity scores for individual samples. Our analysis showed that *DiffRank* and *GSVA* generally perform better than *PLAGE* and *Z-Score*. This suggests that incorporating expression of non-member genes could help better characterize pathway activities than approaches using member genes alone. In addition, both *DiffRank* and *GSVA* adopt a ranking-based strategy to calculate pathway activity for individual samples. Such ranking-based pathway activity is computable for single sample with gene expression profile, which makes it very straightforward to perform prediction on new samples, i.e. the N-of-1 situations in precision medicine. However, other approaches to compute pathway activity could be used as well. For example, pathways topology have been used to improve pathway enrichment analysis [31]. In our context, pathway structures could also be utilized to help define the importance of genes to improve the pathway activity scoring.

In this study, Elastic net was used to build the predictive models of drug response. We recognize that other machine learning algorithms (i.e. random forest, neural networks) could also be tested in an attempt to improve the prediction of some of the drugs that display poor correlations with IC50 values (data not shown). Prediction performance could also be improved by including additional -omics data types, such as copy number, methylation, etc. Finally, this study was based on canonical pathways, which involve only genes curated in pathway databases. More gene-sets could be assembled or derived from molecular interaction network, such as densely connected sub-networks or downstream target genes of regulators (i.e. transcription factors) etc. Such molecular networks could cover more genes that are involved in drug responses to improve the accuracy of the predictive models.

## Conclusion

We developed a pathway-based modelling strategy to predict drug response of cancer cells. The results show that pathway-based models achieve comparable or even better drug response prediction than gene-based models. Moreover, we have shown that pathway-based models recapitulate known drug response mechanisms for majority of drugs. Pathway-based models could serve as an effective alternative to gene-based models for predicting drug sensitivities of cancer cells.

## Additional files

Additional file 1: Metrics of pathway member genes and their correlations. (XLSX 127 kb)

Additional file 2: Target genes of CCLE drugs. (XLSX 16 kb)

Additional file 3: Coefficients of pathways in elastic net models (based on DiffRank) for each CCLE drug. Pathways are indicated when their member genes are related to CCLE drugs. (XLSX 64 kb)

Additional file 4: Coefficients of genes in elastic net models for two drugs. (XLSX 13 kb)

Additional file 5: Pathway map for anti-apoptotic action of ErbB2 in breast cancer. (PNG 1088 kb)

Additional file 6: Pathway map for mytomycin action. (PNG 853 kb)

## Abbreviations

CCLE: Cancer Cell Line Encyclopedia
GDSC: Genomic Drug Sensitivity of Cancer
GSEA: Gene Set Enrichment Analysis
GSVA: Gene Set Variation Analysis
MACC: Median of Absolute Correlation Coefficients
MSE: Mean Square Error
