# Supplementary figures and images for: Predict drug sensitivity of cancer cells with pathway activity inference
Source: BMC Med Genomics. 2019 Jan 31;12(Suppl 1):15. doi: 10.1186/s12920-018-0449-4 (PMC6357358; doi:10.1186/s12920-018-0449-4)

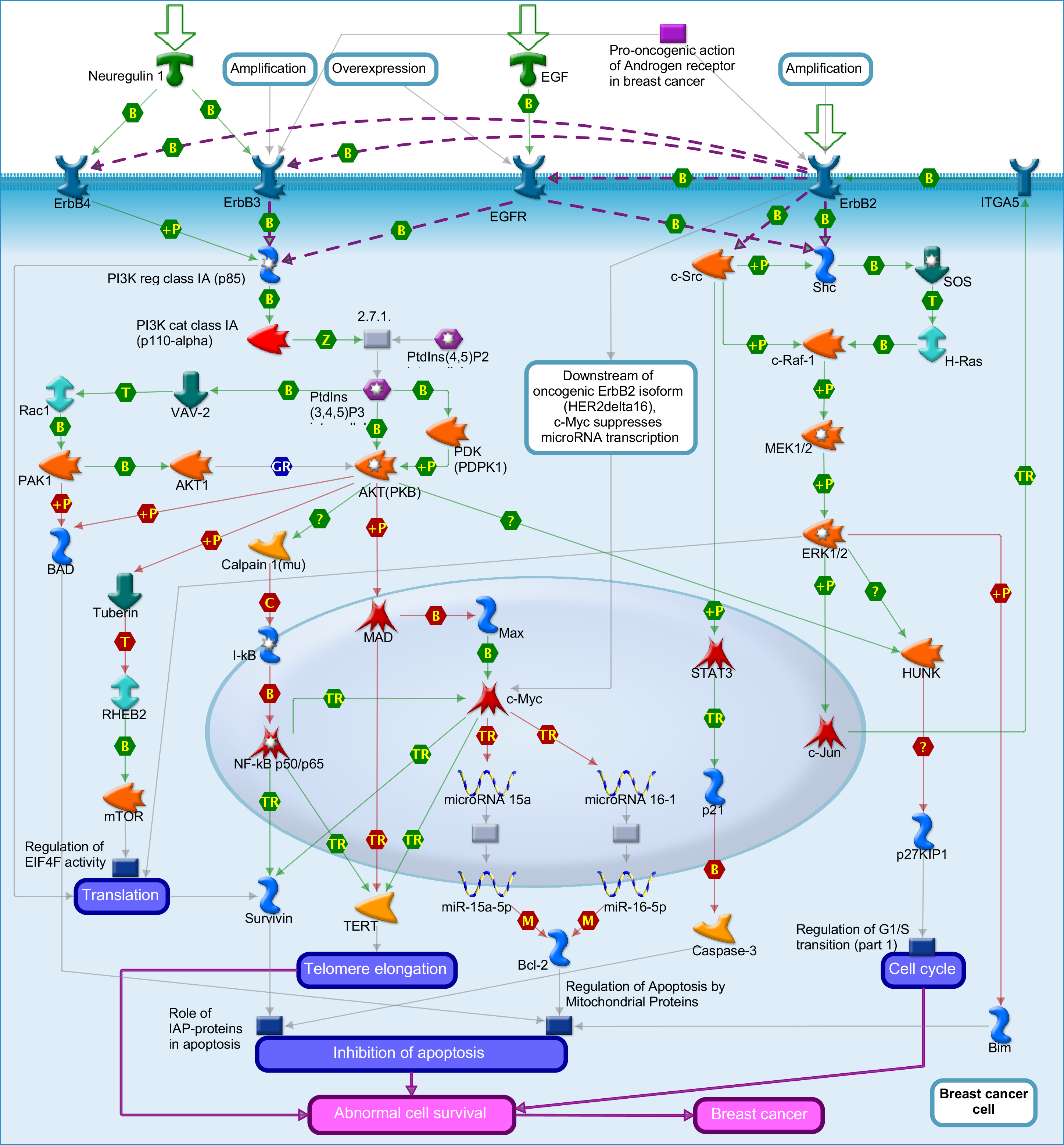

Supplement: Supplementary file 5 — Pathway map for anti-apoptotic action of ErbB2 in breast cancer. (PNG 1088 kb) [file 12920_2018_449_MOESM5_ESM.png]

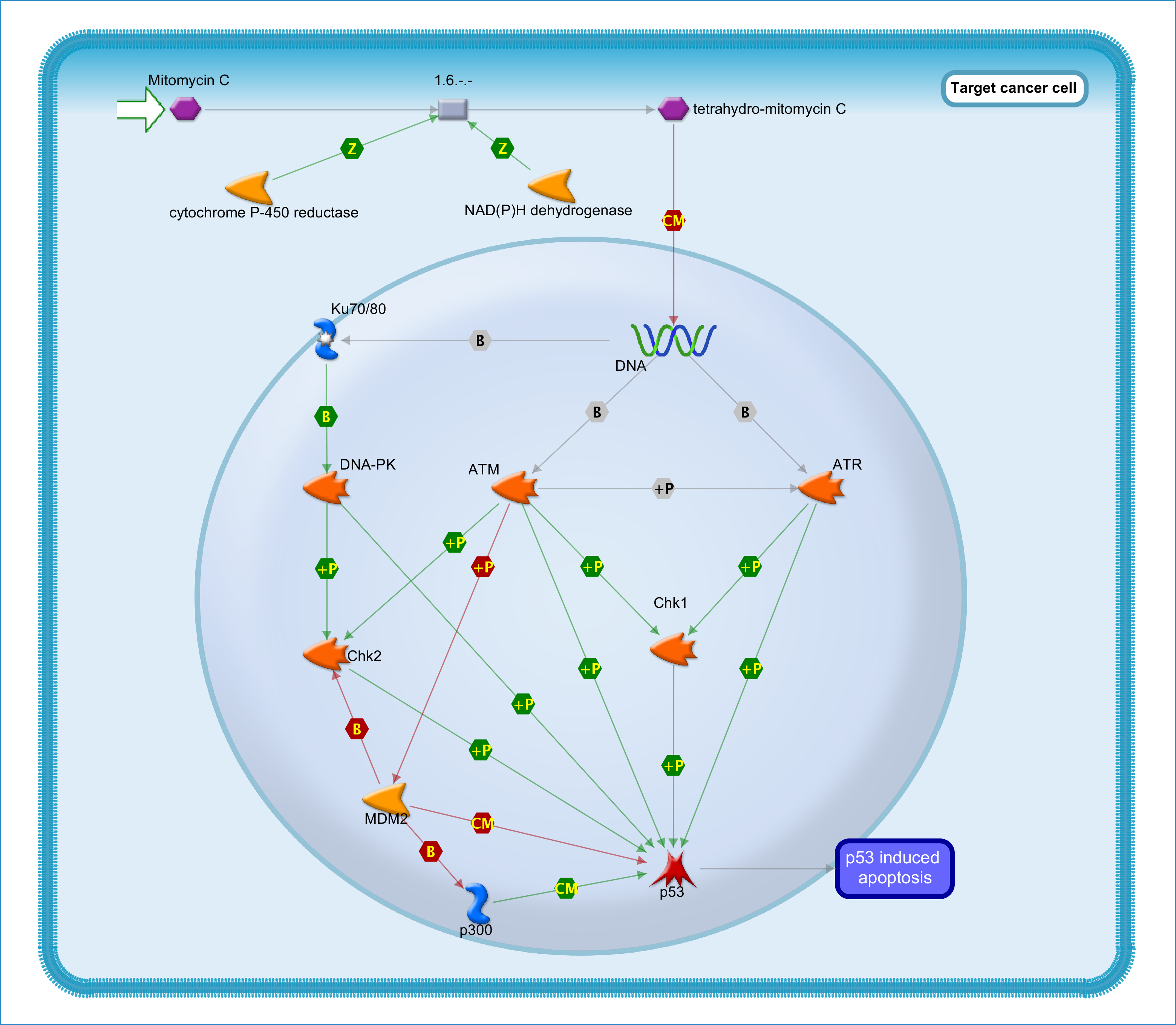

Supplement: Supplementary file 6 — Pathway map for mytomycin action. (PNG 853 kb) [file 12920_2018_449_MOESM6_ESM.png]
